# Let's Talk About Sex: Breaking Down Barriers to Care in a Community Learning Disability Team

**DOI:** 10.1192/bjo.2024.397

**Published:** 2024-08-01

**Authors:** Liam Embliss, Zoe Melrose, Laura Dunn

**Affiliations:** 1 North East London NHS Foundation Trust, London, United Kingdom; 2 South West London and St George's Mental Health NHS Trust, London, United Kingdom; 3 East London NHS Foundation Trust, London, United Kingdom

## Abstract

**Aims:**

We aimed to improve the care for people with intellectual disabilities (PWID) presenting to a community learning disability service (CLDT) with health needs relating to sexual wellbeing, sexuality, and/or gender identity (SSGI). A QI framework was used, focussing on staff education and service development.

We hypothesised that there would be a lack of confidence and staff knowledge around SSGI issues in PWID. We suggested that challenges exist because discussing sex in PWID still feels taboo.

PWID have the same sexual needs as those without any disability. Historically, this population have been discouraged from expressing their sexuality due to certain attitudes, fears, and prejudices. Stigmatising views have included PWID being viewed as asexual or conversely posing a risk of sexual violence, despite evidence showing that they are more vulnerable to sexual abuse. Important issues around capacity and understanding consent highlight the importance of psychosexual education for patients and carers.

Carers and health care professionals are key in educating and supporting PWID, however, our disinclination towards discussing SSGI openly can have unintended negative effects on the wellbeing of our patients. These issues are therefore paramount to understand and address.

**Methods:**

Patient-facing staff in a London CLDT were surveyed, and staff focus groups held, to understand attitudes towards SSGI in PWID. Staff knowledge of local services was also explored. Using thematic analysis, we identified both staff and service development needs and devised a set of interventions to address these.

Four educational interventions for staff were developed and evaluated using QI methodology. Interventions included bitesize teaching, externally commissioned training, and resource packs.

**Results:**

Thematic analysis identified a number of barriers to delivering SSGI care, particularly staff's low confidence and a lack of training. Following the four educational interventions, average staff confidence to discuss SSGI increased from 55% to 77%.

Staff responses indicated a lack of SSGI services for PWID locally. In response to this, the QI team, service leads and management agreed upon various service development ideas. These include upskilling specific staff to become SSGI leads; auditing the CLDT caseload to understand the SSGI issues in our population; and trialling a clinical sexology service for a small subset of patients.

**Conclusion:**

A QI approach to staff education demonstrated clear benefit, with staff more confident to address the SSGI needs of PWID. Combined with sustainable service improvement ideas, this can improve patient care.

